# Endocytic uptake of SARS-CoV-2: the critical roles of pH, Ca^2+^, and NAADP

**DOI:** 10.1093/function/zqaa003

**Published:** 2020-06-05

**Authors:** Ole H Petersen, Oleg V Gerasimenko, Julia V Gerasimenko

**Affiliations:** School of Biosciences, Cardiff University, Cardiff CF10 3AX, Wales, UK

It has recently been shown that SARS-CoV-2 enters cells via receptor-mediated endocytosis, as expected from previous studies of other coronaviruses.^1^ ACE2 is the receptor for SARS-CoV-2, which unlocks access to the endocytic uptake of the virus.^2^ It has also been demonstrated that a two-pore channel (TPC2) is critically important for SARS-CoV-2 entry into cells.^1^ From earlier studies, we know that TPCs are channels in the endo-lysosomal system through which Ca^2+^ is released when activated by the intracellular messenger nicotinic acid adenine dinucleotide phosphate (NAADP).^3^ The new findings on SARS-CoV-2 entry^1^ link two hitherto completely separate research fields, namely physiological Ca^2+^ signaling and virology, opening up intriguing and important opportunities for future work on the cellular pathophysiology of COVID-19.

Endocytosis is a process whereby substances in the extracellular fluid can be taken into cells without endangering the integrity of the plasma membrane (Figure 1). Endosomes have an acid interior due to the operation of a proton pump that can be very specifically inhibited by bafilomycin (Figure 1). Ou et al.^1^ have shown that SARS-CoV-2 entry is completely blocked when the proton pump is inhibited by 100 nM bafilomycin A, a concentration of the inhibitor that has previously been shown to prevent endosomal acidification in different cell types (Figure 1).^4^^,^^6^

**Figure 1.**
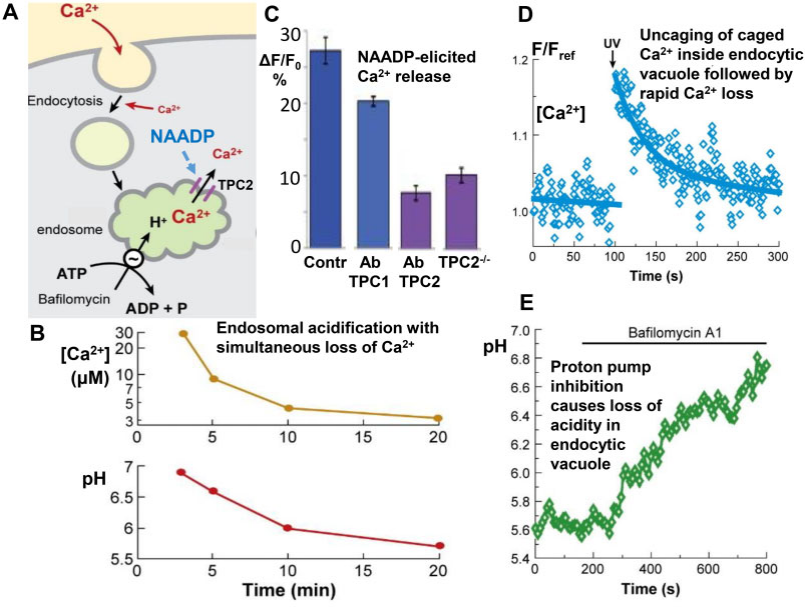
The link between endosomal acidification and loss of Ca^2+^ from endosomal organelles. (A) Simplified sketch of endosomal Ca^2+^ uptake, acidification of the endosome by the bafilomycin-sensitive proton pump and leak of Ca^2+^ from the endosome, via NAADP-sensitive TPCs. (B) Time course of endosomal acidification and Ca^2+^ loss in fibroblasts. (C) Effects of antibodies against TPC1 and TPC2 as well as deletion of TPC2 on NAADP-elicited Ca^2+^ release from permeabilized pancreatic acinar cells. (D) Rapid loss of Ca^2+^ from pancreatic acinar endocytic vacuole following sudden elevation of [Ca^2+^] inside vacuole due to Ca^2+^ uncaging. (E) Rapid increase in pH of pancreatic acinar endocytic vacuole after blockage of proton pump by 100 nM bafilomycin A1. Data in (B) are from Gerasimenko et al. 1998,^4^ in (C) from Gerasimenko et al. 2015^5^ and in (D) and (E) from Sherwood et al. (2007).^6^

It is particularly intriguing that pharmacological inhibition of TPC2, with tetrandrine, markedly reduced cellular entry of SARS-CoV-2.^1^ This finding is similar to what has been shown in much more detail with regard to the entry of the Ebola virus.^7^ Deletion of TPC1 or TPC2 resulted in marked inhibition of virus entry.^7^ Furthermore, several known inhibitors of NAADP-elicited Ca^2+^ release from acid stores, for example, Ned-19 and tetrandrine, also severely reduced entry of this virus.^7^ From earlier Ca^2+^ signaling studies, it is known that deletion of TPC2 markedly reduced NAADP-elicited Ca^2+^ release in permeabilized cells, as did an antibody to TPC2 (Figure 1). Ca^2+^ release mediated by NAADP in intact cells was also markedly inhibited by Ned-19.^5^

Endocytic acidification occurs at the same time as Ca^2+^ is lost from endosomes (Figure 1). The fluid taken into cells by endocytosis has, of course, the normal concentration of free Ca^2+^ found in the extracellular fluid, namely ∼1mM. However, as seen in Figure 1, at the earliest time points allowing assessment of endosomal [Ca^2+^], the concentration had already dropped to ∼30 μM, which is still markedly higher than the level in the cytosol (∼0.1 μM). The endosomal [Ca^2+^] then declined further to ∼4 μM in late endosomes (Figure 1). Endosomal acidification and loss of Ca^2+^ are interlinked. Blockade of the proton pump by bafilomycin prevented release of Ca^2+^ from the endosomes, with endosomal [Ca^2+^] remaining high.^4^ If [Ca^2+^] in the early endosomes was kept low, by reducing the extracellular [Ca^2+^] to less than 300 μM, acidification did not occur.^4^ The mechanism underlying the linkage between Ca^2+^ and H^+^ movements is not fully understood, but the fact that the uptake of H^+^ into endosomes occurs simultaneously with release of Ca^2+^, and that the two processes are interdependent, means that it is difficult to determine whether inhibition of virus entry by blockade of the proton pump or by TPC inhibition^1^^,^^7^ is primarily due to the high pH or to the relatively high [Ca^2+^] in the endosomes, or to a mixture of both.

Since TPCs are NAADP-sensitive channels, it is important to understand the mechanism by which NAADP is formed. The CD38 enzyme responsible for NAADP generation is an ecto-enzyme that is internalized by endocytosis,^8^ and requires an acid environment to perform its task.^8^ In pancreatic acinar cells, the process of NAADP formation and the mechanism of action of this messenger have been investigated in some detail.^5^^,^^8^ In this cell type, there is evidence showing that it is in the acidic endosomes that NAADP generation takes place.^8^ Furthermore, the initial Ca^2+^ release triggered by NAADP may depend on actions of this messenger on both endosomes and lysosomes.^5^ At least part of the effect of proton pump inhibition on virus entry^1^^,^^7^ could therefore be explained by lack of NAADP formation^8^ and action, thereby preventing TPC activation.^5^

SARS-CoV-2 is not only entering cells in the respiratory tract,^2^ but is also taken up in the central nervous system, where it can cause serious damage,^9^ as well as in the gastro-intestinal tract, including the pancreas.^10^ From a mechanistic perspective, it may be of particular interest to study virus uptake in pancreatic acinar cells, since in these cells there is clear evidence for a physiological role of NAADP.^5^ In this context, it is interesting that COVID-19 can be associated with acute pancreatitis and that ACE2 is present in the pancreas.^10^ In acute pancreatitis, a human disease in which the pancreas digests itself, there is a secretion defect and large endocytic vacuoles, which are different from endosomes and post-exocytic in nature, appear in the acinar cells.^6^ Nevertheless, these vacuoles have functional features in common with endosomes. When NAADP is generated by hormonal stimulation, the vacuole membrane is very permeable to Ca^2+^, as demonstrated by the rapid loss of Ca^2+^ from vacuoles following a sudden increase in [Ca^2+^] elicited by uncaging of caged Ca^2+^ by UV light (Figure 1D). The endocytic vacuoles are also acidic, with a pH similar to that found in endosomes, and bafilomycin A1—the very specific proton pump blocker—rapidly increased pH inside the vacuoles (Figure 1E). The endocytic vacuoles may provide an additional and important entry pathway for viruses, including SARS-CoV-2. Furthermore, the post-exocytic vacuoles contain large amounts of many different proteases,^6^ which could also play a role in SARS-CoV-2 infection.

Exocrine pancreatic secretion is principally controlled by the neurotransmitter acetylcholine (ACh) and the hormone cholecystokinin (CCK). Both agents elicit secretion by mobilizing intracellular Ca^2+^, which subsequently triggers Ca^2+^ entry from the extracellular solution. CCK primarily evokes Ca^2+^ release mediated by NAADP acting not only on TPCs—but also on ryanodine receptors—in a complex arrangement involving endosomes and lysosomes.^5^ In contrast, ACh primarily causes intracellular Ca^2+^ release mediated by inositol trisphosphate (IP_3_), acting on IP_3_ receptors principally located in the endoplasmic reticulum.^5^ Given that endosomal acidification depends on endosomal Ca^2+^ release,^4^ and that the entry of SARS-CoV-2 and Ebola virus is abolished or severely reduced when the proton pump is blocked or when TPCs are inhibited,^1^^,^^7^ it would be of considerable interest to know if and how NAADP-dependent Ca^2+^ signaling influences SARS-CoV-2 entry. Taking advantage of the co-existence in pancreatic acinar cells of two different Ca^2+^ signaling mechanisms, activated by separate receptor pathways, it would seem possible to provide well-defined test and control conditions in such studies.

We conclude that there is evidence for the involvement of acidic endosomes and TPC-mediated Ca^2+^ release from the endo-lysosomal system in both SARS-CoV-2 entry and NAADP-mediated Ca^2+^ signaling. The pancreatic acinar cells may prove to be a particularly useful system for exploring the relationship between these phenomena.

## Conflicts of interest statement

None declared.

## Funding

Experimental work in the authors’ laboratory was supported by grants from the UK’s Medical Research Council (MR/J002771/1 and G19/22/2).
